# CT-M8 Mice: A New Mouse Model Demonstrates That Basophils Have a Nonredundant Role in Lupus-Like Disease Development

**DOI:** 10.3389/fimmu.2022.900532

**Published:** 2022-06-29

**Authors:** John Tchen, Quentin Simon, Léa Chapart, Christophe Pellefigues, Hajime Karasuyama, Kensuke Miyake, Ulrich Blank, Marc Benhamou, Eric Daugas, Nicolas Charles

**Affiliations:** ^1^ Université Paris Cité, Centre de Recherche sur l’Inflammation, Institut National de la Santé Et de la Recherche Médicale (INSERM) UMR1149, Centre National de la Recherche Scientifique (CNRS). EMR8252, Faculté de Médecine site Bichat, Paris, France; ^2^ Université Paris Cité, Laboratoire d’Excellence INFLAMEX, Paris, France; ^3^ Inflammation, Infection and Immunity Laboratory, TMDU Advanced Research Institute, Tokyo Medical and Dental University (TMDU), Tokyo, Japan; ^4^ Service de Néphrologie, Hôpital Bichat, Assistance Publique-Hôpitaux de Paris, Paris, France

**Keywords:** basophils, mouse model, systemic lupus erythematosus, lupus nephritis, autoimmunity, TH2

## Abstract

Tissue-specific mouse models are essential tools to decipher the role of each cell compartment and/or their expressed genes in the pathophysiology of diseases. Here, we describe a new knock-in mouse model allowing expression of both the fluorescent protein tdTomato and the CRE recombinase selectively in the basophil compartment under the control of the Mcpt8 gene. These “CT-M8” mice did not show any abnormalities in their peripheral distribution of major immune cell populations nor their basophil function. CT-M8 mice allowed the identification of basophils by immunofluorescence and flow cytometry and basophil-specific Cre-mediated floxed gene deletion. Breeding of our CT-M8 mice with the *ROSA26^flox-stop-DTA^* mice led to the generation of basophil-deficient mice with no detectable abnormalities in other cell compartments. These mice were then used to document basophil involvement in systemic lupus erythematosus (SLE) pathophysiology since we previously reported by transient depletion of these cells during the course of an ongoing disease that they support and amplify autoantibody production in two distinct lupus-like mouse models (*Lyn^−/−^* and pristane-induced). Here, constitutive basophil deficiency prevented pristane-induced lupus-like disease development by limiting autoantibody titers and renal damages. Therefore, basophils have a nonredundant role in pristane-induced lupus-like disease and are involved in both its induction and amplification. This CT-M8 new mouse model will allow us to finely decipher the role of basophils and their expressed genes in health and disease.

## Introduction

Systemic lupus erythematosus (SLE) is an autoimmune disease (AID) affecting mainly women of child-bearing age. SLE can affect different organs, such as skin, lungs, joints, or kidneys. Lupus nephritis is one of the most severe organ manifestations of SLE. It affects 25% to 60% of SLE patients, and about 20% of these patients develop a life-threatening end-stage renal disease (1). SLE is characterized by the presence in the patient’s blood of autoreactive antibodies of several isotypes, mainly raised against nuclear antigens such as double-stranded DNA (dsDNA) or ribonucleoproteins (RNPs) (2). Once conjugated to autoantigens and complement factors, the formed immune complexes (ICs) can deposit in target organs where they induce inflammation, leading to organ injury and consequent organ dysfunction. ICs also induce Fc receptor-dependent activation of innate immune cells such as plasmacytoid dendritic cells (pDCs), neutrophils, macrophages, and basophils, which all contribute to an amplification loop of autoantibody production and disease activity (1, 3).

We previously reported that basophils accumulate in secondary lymphoid organs (SLOs) during lupus pathogenesis through mechanisms dependent on IgE, IL-4, prostaglandin D2 (PGD2), and CXCR4. There, they promote autoreactive plasma cell accumulation and consequent autoantibody production (4–6). These findings were validated in several human SLE patient cohorts and multiple lupus-like mouse models. Among these models, basophils were shown to contribute to the pristane-induced lupus-like disease (PIL) (7). In mice with established disease, basophil depletion dampened disease activity by reducing short-lived plasma cell number, serum autoantibody titers, IC deposition in glomeruli, and the proinflammatory environment in the kidneys (4, 6, 7), identifying basophils as promising therapeutic targets in SLE. However, whether basophils are involved in the primary development of the disease remains unknown.

Here, we report the generation of a new mouse model called CT-M8 (*Mcpt8^tm1Ics^*) that allows the expression of the CRE recombinase (CRE) and the tandem Tomato (tdT) fluorescent protein downstream of the gene of the basophil-specific protease mast cell protease 8 (*Mcpt8*) (8). This model enabled convenient basophil detection by fluorescent microscopy and flow cytometry as well as basophil-specific floxed gene deletion. Breeding of these CT-M8 mice with the ROSA-DTA mice (expressing a loxP site-flanked stop cassette upstream of the gene encoding diphtheria toxin A under the control of the Rosa26 promoter [B6.129P2-Gt(ROSA)26Sortm1(DTA)Lky/J)] (9) allowed the generation of constitutive basophil-deficient mice (*Mcpt8^CT/+^ Rosa26^DTA/+^*). In the PIL model, basophil deficiency prevented the accumulation of short-lived plasma cells in SLOs as well as the detection of anti-RNP autoantibodies and IC deposits in the glomeruli of the mice 8 weeks after pristane injection. Overall, this new model of basophil-deficient mice allowed showing that basophils have a nonredundant role in PIL development and might be mandatory in the SLE-like disease course to reach a pathogenic threshold.

## Material and Methods

### Generation of *Mcpt8^tm1Ics^* or CT-M8 Mice

**Figure 1:**
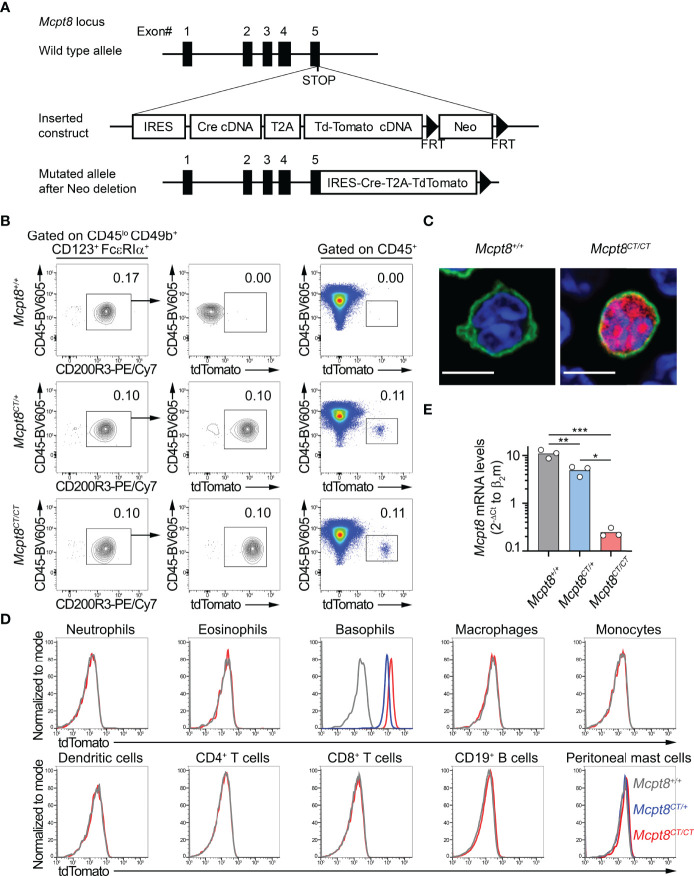
The CT-M8 mice: a new basophil-specific mouse model. **(A)** Schematic representation of Mcpt8-Cre-tdT knock-in targeting constructs to generate the CT-M8 (*Mcpt8^tm1Ics^*) mice (see *Methods*) where Cre and tandem tomato fluorescent protein are expressed under the control of the basophil-specific Mcpt8 promoter. **(B)** Fluorescence-activated cell sorter (FACS) gating strategy showing tdTomato expression in spleen basophils from *Mcpt8^CT/CT^* and *Mcpt8^CT/+^* mice but not in wild-type animals (*Mcpt8^+/+^*). **(C)** Basophil-specific tdTomato expression (red) imaged by confocal microscopy on splenocytes from *Mcpt8^+/+^* (left) and *Mcpt8^CT/CT^* (right) mice, stained with anti-IgE antibody (green) and DNA staining (DAPI, blue). Scale bar, 5 µm. Larger fields are shown in **Supplementary Figure S1**. **(D)** Expression of the tdTomato assessed by flow cytometry in the indicated spleen cell types (except for peritoneal mast cells, which are from the peritoneal cavity) defined as described in **Table 1** in *Mcpt8^+/+^* (grey lines), *Mcpt8^CT/+^* (blue lines), and *Mcpt8^CT/CT^* (red lines). One representative set of data out of 4 per genotype is shown. **(E)** Mcpt8 mRNA expression levels were assessed by RT-quantitative PCR on mRNA purified from FACS-sorted bone marrow primary basophils from mice of the indicated genotypes (see *Methods*). Data are expressed as the mean of the 2^−ΔCt^ values of Mcpt8 relative to the β_2_-microglobulin Ct. Individual values are shown inside bars. Statistical analyses were done by one-way ANOVA followed by Tukey’s multiple comparisons test. ^*^ *p* < 0.05; **p < 0.01; ^***^ *p* < 0.001.

The C57BL/6 Mcpt8-Cre-tdTomato or *Mcpt8^tm1Ics^* or “CT-M8” mice were generated by the Institut Clinique de la Souris (Illkirch, France) from the Phenomin consortium. Briefly, a cassette containing an internal ribosome entry site (IRES), the cDNA encoding the CRE recombinase, a viral Thosea asigna virus 2A (T2A) sequence, and the fluorescent protein tandem Tomato or “tdT” was knocked in together with a Neo resistance (Neo^r^) cassette flanked by two FRT sites after the stop codon of the Mcpt8 gene. Chimeric mice were generated by the injection of mutant ES cells into C57BL/6 blastocysts and cross-bred with Flp deleter C57BL/6N females (10) to obtain Neo^r^-deleted *Mcpt8^tm1lcs^* mice (**Figure 1A**).

### Mice

C57BL/6J wild-type mice were purchased from Charles River Laboratories (Ecully, France). The Rosa26-loxP-Stop-loxP-DTA C57BL/6J $\left(R_{26}^{DTA/DTA}\text{ or }R_{26}^{DTA/+}\right)$ mice (9) were purchased from The Jackson Laboratory through Charles River Laboratories. *Il4^fl/fl^* C57BL/6J mice were previously described (11). For all experiments, WT, *Mcpt8^CT/CT^*, *Mcpt8^CT/+^*, *Mcpt8^CT/+^* x *Il4^fl/fl^*, *Il4^fl/fl^*, $R_{26}^{DTA/DTA}$ , and $Mcpt8^{CT/+}\times R_{26}^{DTA/+}$ mice were used on a C57BL/6J, C57BL/6N, or C57BL/6JxN mixed background.

For phenotyping experiments, mice from both sexes in equal proportions were used between 8 and 15 weeks of age. For pristane-induced lupus-like (PIL) disease experiments, 8-week-old female mice of the indicated genotype received a single intraperitoneal injection of 500 µl of pristane (Sigma-Aldrich, Burlington, MA, USA) or phosphate-buffered saline (PBS, Gibco) as a control and were sacrificed 8 weeks later. Mice were maintained under specific pathogen-free conditions in our animal facilities. The study was conducted in accordance with the French and European guidelines and approved by the local ethics committee, comité d’éthique Paris Nord N°121 and the Ministère de l’enseignement supérieur, de la recherche et de l’innovation under the authorization number APAFIS#14115.

### Mouse Sample Processing

Mice were euthanized in a regulated CO_2_ chamber. Blood was harvested with a heparinized syringe with a 25-G needle by cardiac puncture. Peritoneal lavages were realized by injecting 5 ml of PBS and 2 ml of air into the peritoneal cavity. After 50 rotations, the peritoneal wall was opened and peritoneal lavage was harvested with a pipette. Cervical, axillary, and inguinal lymph nodes were harvested and pooled. Bone marrow was extracted from both femurs, and the spleen was harvested. The left kidney was embedded in OCT (CellPath, Newton, UK) and snap-frozen in liquid nitrogen and kept at −80°C until immunofluorescence analysis. Single-cell suspensions from blood, bone marrow, peritoneal lavage, spleen, or peripheral lymph nodes (LNs) (pooling inguinal, axillary, and cervical LNs) were prepared by mechanical disruption over a 40-μm cell strainer (Falcon, Corning, Corning, NY, USA) when necessary. Blood was centrifuged at 300×*g* for 10 min at room temperature, and plasma was harvested for further analysis. The specimens were incubated for 5 min at room temperature in ACK lysing buffer (150 mM of NH_4_Cl, 12 mM of NaHCO_3_, 1 mM of EDTA, pH 7.4) and neutralized with twice the volume of PBS, and cells were centrifuged (500×*g*, 5 min). This step was repeated three times for blood samples. Cell concentrations were determined with a hemacytometer, and 1–5 million cells per point were used for fluorescence-activated cell sorting (FACS) staining (see below).

### Flow Cytometry

Unspecific antibody-binding sites were saturated with a blocking buffer containing 10 µg/ml of anti-CD16/CD32 antibody clone 2.4G2 (BioXcell, Lebanon, NH, USA), and 100 µg/ml of polyclonal rat IgG, polyclonal mouse IgG, and polyclonal Armenian Hamster IgG (Innovative Research, Inc., Novi, MI, USA) in FACS buffer (PBS, 1% bovine serum albumin, 1 mM EDTA, and 0.05% sodium azide). Mouse cells were stained in optimized concentration of fluorophore-conjugated monoclonal antibodies (clone) targeting CD45 (30-F11), FcϵRIα (MAR1), CD49b (DX5), IA/IE (M5/114.2), CD200R1 (OX-110), CD200R3 (Ba13), TCRβ (KT3.1.1), CD4 (RM4-4), CD8α (53-6.7), CD11b (M1/70), F4/80 (BM8), CD11c (N418), CD317 (927), Ly6G/Ly6C (Gr1, RB6-8C5), CD19 (6D5), CD117 (2B8), and CD138 (281-2), all from BioLegend, San Diego, CA, USA; and IL-4 (11B11) from BD Biosciences, Franklin Lakes, NJ, USA; IL-6 (MP5-20F3), IL-13 (eBio13A), and CD123 (5B11) from ThermoFisher Scientific, Waltham, MA, USA. Basophils were defined as CD45^lo^ CD3^−^ CD19^−^ CD117^−^ CD200R3^+^ CD49b^+^ FcϵRIα^+^ CD123^+^ cells among CD45^+^ viable singlets. Peritoneal mast cells were defined as CD45^+^ CD3^−^ CD19^−^ F4/80^−^ FcϵRIα^+^ CD117^+^. Other cells were defined as indicated in **Tables 1**, **2**. For all flow cytometry experiments, dead cells were stained in PBS with GHOST 510 viability dye (TONBO, San Diego, CA, USA) and excluded from the analysis. For intracellular cytokine staining, cells were fixed for 30 min at 4°C with fixation buffer and staining was realized in intracellular staining permeabilization wash buffer following the manufacturer’s instructions (both buffers from BioLegend, San Diego, CA, USA). Flow cytometry acquisition was realized using a Becton Dickinson 5-laser LSR Fortessa X-20 and data analysis using Flowjo vX (Treestar, BD Biosciences, Franklin Lakes, NJ, USA). For assessment of the CD200R1 expression level, the ratio of the geometric mean fluorescence intensity (gMFI) of CD200R1 to the fluorescence minus one (containing the isotype control) gMFI was calculated and expressed in arbitrary units (A.U.).

**Table 1:** Immune cell populations in *Mcpt8^+/+^*, *Mcpt8^CT/+^*, and *Mcpt8^CT/CT^* mice.

Population	Defined in CD45^+^ living singlets as cells	*Mcpt8^+/+^* (*n* = 4)		*Mcpt8^CT/+^* (*n* = 4)			*Mcpt8^CT/CT^* (*n* = 4)		
		Mean	SD	Mean	SD	*p*-value to +/+	Mean	SD	*p*-value to +/+
**Spleen**									
Basophils	CD45^lo^ CD49b^+^ FcϵRIα^+^ CD123^+^ CD200R3^+^	0.18	0.0483	0.1165	0.0275	*0.8284*	0.224	0.2574	*0.9127*
tdT^+^ cells	CD45^+^ tdT^+^	0.0010	0.0007	0.1275	0.0330	***<0.0001***	0.1033	0.0115	***0.0006***
CD19^+^ B cells	CD45^+^CD19^+^	52.75	4.133	51.18	1.258	*0.6875*	52.15	1.515	*0.9451*
CD4^+^ T cells	CD45^+^ TCRβ^+^ CD4^+^ CD8^–^	19.18	1.497	19.58	2.232	*0.9871*	17.65	5.786	*0.8316*
CD8^+^ T cells	CD45^+^ TCRβ^+^ CD4^−^ CD8^+^	11.5	1.481	10.98	0.6898	*0.8056*	10.64	1.218	*0.5755*
Neutrophils	CD45^+^ TCRβ^−^ CD19^−^ Gr1^hi^ CD11b^hi^	2.548	0.6401	2.895	0.633	*0.681*	3.113	0.429	*0.3867*
Macrophages	CD45^+^ TCRβ^−^ CD19^−^ Neutro^−^ CD11b^lo^ F4/80^hi^	1.015	0.6523	0.98	0.6606	*0.9963*	0.6725	0.4869	*0.7122*
Eosinophils	CD45^+^ TCRβ^−^ CD19^−^ Neutro^−^ Macro^−^ CD11b^+^ SSC^hi^	0.475	0.1731	0.5525	0.4801	*0.9308*	0.5275	0.1198	*0.9675*
pDCs	CD45^+^ TCRβ^−^ CD19^−^ Neutro^−^ Macro^−^ Eosino^−^ CD11c^+^ PDCA1^+^	0.4625	0.3356	0.45	0.3982	*0.9987*	0.395	0.3534	*0.9628*
DCs	CD45^+^ TCRβ^−^ CD19^−^ Neutro^−^ Macro^−^ Eosino^−^ pDC^−^ CD11c^+^ IA-IE^+^	1.303	0.1406	1.355	0.2063	*0.8999*	1.115	0.152	*0.3059*
Monocytes	CD45^+^ TCRβ^−^ CD19^−^ Neutro^−^ Macro^−^ Eosino^−^ pDC^−^ DC^−^ CD11b^+^	1.618	0.6074	1.828	0.2952	*0.8218*	1.95	0.5311	*0.6636*
**Bone marrow**									
Basophils	CD45^lo^ CD49b^+^ FcϵRIα^+^ CD123^+^ CD200R3^+^	0.77	0.0828	0.7275	0.1209	*0.8433*	0.6275	0.1135	*0.199*
tdT^+^ cells	CD45^+^ tdT^+^	0.0034	0.0036	0.7475	0.1087	***<0.0001***	0.6525	0.1115	***<0.0001***
Neutrophils	CD45^+^ TCRβ^−^ CD19^−^ Gr1^hi^ CD11b^hi^	60.9	5.363	65.88	3.82	*0.4056*	61.8	6.185	*0.9678*
Eosinophils	CD45^+^ TCRβ^−^ CD19^−^ Neutro^−^ Macro^−^ CD11b^+^ SSC^hi^	4.138	1.325	4.145	2.126	*>0.9999*	4.228	1.646	*0.997*
**Blood**									
Basophils	CD45^lo^ CD49b^+^ FcϵRIα^+^ CD123^+^ CD200R3^+^	0.3733	0.2515	0.2925	0.1565	*0.8186*	0.2725	0.1162	*0.7355*
tdT^+^ cells	CD45^+^ tdT^+^	0.0056	0.0026	0.2875	0.1735	***0.021***	0.2525	0.1112	***0.04***
Neutrophils	CD45^+^ TCRβ^−^ CD19^−^ Gr1^hi^ CD11b^hi^	23.8	6.483	27.45	6.675	*0.6468*	22.2	3.093	*0.9165*
Eosinophils	CD45^+^ TCRβ^−^ CD19^−^ Neutro^−^ Macro^−^ CD11b^+^ SSC^hi^	2.005	0.8843	1.948	0.9925	*0.9954*	2.1	0.7896	*0.9876*
**Peritoneal cavity**									
Mast cells	CD45^+^ F4/80^−^ FcϵRIα^+^ CD117^+^	2.313	0.640	2.378	0.726	*0.9879*	2.268	0.218	*0.9938*

Proportions of immune cell populations among CD45^+^ cells in the indicated compartments were determined by flow cytometry. Cells were defined following the indicated gating strategy; 8- to 15-week-old mice were analyzed. Statistical analysis was done by one-way analysis of variance (ANOVA) followed by Tukey’s multiple comparison test. p-values indicated are from comparison to the Mcpt8^+/+^ group. SD, standard deviation.

Bold/Italic values in Table 1 and 2 are the statistically significant values (p<0.05).

**Table 2:** Immune cell populations in basophil-deficient mice.

Population	Defined in CD45^+^ living singlets as cells	*Mcpt8^CT/+^* (*n* = 8–12)		*Mcpt8^CT/+^R26-Stop^fl/WT^-DTA* (*n* = 5–12)			*Mcpt8^+/+^ R26-Stop^fl/fl^-DTA* (*n* = 5–12)		
		Mean (% of CD45^+^)	SD	Mean (% of CD45^+^)	SD	*p*-value to *Mcpt8^CT/+^*	Mean (% of CD45^+^)	SD	*p*-value to *Mcpt8^CT/+^*
**Spleen**									
Basophils	CD45^lo^ CD49b^+^ FcϵRIα^+^ CD123^+^ CD200R3^+^	0.1258	0.032	0.0026	0.001	***<0.0001***	0.1586	0.055	0.1799
tdT^+^ cells	CD45^+^ tdT^+^	0.13	0.040	0.0013	0.002	***<0.0001***	0.0010	0.000	***<0.0001***
CD19^+^ B cells	CD45^+^CD19^+^	40.01	13.67	45.28	11.14	*0.6382*	36.08	7.167	*0.8221*
CD4^+^ T cells	CD45^+^ TCRβ^+^ CD4^+^ CD8^−^	24.23	6.822	21.28	4.477	*0.5101*	25.88	2.590	*0.8454*
CD8^+^ T cells	CD45^+^ TCRβ^+^ CD4^−^ CD8^+^	14.98	5.007	13.10	2.290	*0.5696*	16.16	2.624	*0.8378*
Neutrophils	CD45^+^ TCRβ^−^ CD19^−^ Gr1^hi^ CD11b^hi^	3.010	0.922	2.040	1.406	*0.3369*	3.224	1.758	*0.9576*
Macrophages	CD45^+^ TCRβ^−^ CD19^−^ Neutro^−^ CD11b^lo^ F4/80^hi^	1.324	0.759	1.975	1.296	*0.4398*	1.732	0.963	*0.7735*
Eosinophils	CD45^+^ TCRβ^−^ CD19^−^ Neutro^−^ Macro^−^ CD11b^+^ SSC^hi^	0.5400	0.338	0.5125	0.192	*0.9909*	0.8760	0.747	*0.3732*
pDCs	CD45^+^ TCRβ^−^ CD19^−^ Neutro^−^ Macro^−^ Eosino^−^ CD11c^+^ PDCA1^hi^	0.2878	0.315	0.1665	0.116	*0.4971*	0.1098	0.051	*0.3235*
DCs	CD45^+^ TCRβ^−^ CD19^−^ Neutro^−^ Macro^−^ Eosino^−^ pDC^−^ CD11c^+^ IA-IE^+^	1.830	0.738	1.209	0.829	*0.2585*	2.662	0.672	*0.1628*
Monocytes	CD45^+^ TCRβ^−^ CD19^−^ Neutro^−^ Macro^−^ Eosino^−^ pDC^−^ DC^−^ CD11b^hi^	1.938	0.319	1.740	0.939	*0.8224*	2.182	0.483	*0.7945*
**Bone marrow**									
Basophils	CD45^lo^ CD49b^+^ FcϵRIα^+^ CD123^+^ CD200R3^+^	0.7870	0.141	0.0210	0.008	***<0.0001***	0.6957	0.215	*0.3735*
tdT^+^ cells	CD45^+^ tdT^+^	0.8667	0.138	0.0267	0.011	***<0.0001***	0.0080	0.005	***<0.0001***
Neutrophils	CD45^+^ TCRβ^−^ CD19^−^ Gr1^hi^ CD11b^hi^	47.07	4.794	48.05	5.363	*0.9381*	49.84	6.493	*0.7566*
Eosinophils	CD45^+^ TCRβ^−^ CD19^−^ Neutro^−^ Macro^−^ CD11b^+^ SSC^hi^	4.575	1.600	5.323	1.788	*0.5082*	4.542	1.055	*0.9987*
**Blood**									
Basophils	CD45^lo^ CD49b^+^ FcϵRIα^+^ CD123^+^ CD200R3^+^	0.4580	0.234	0.0035	0.003	***<0.0001***	0.5471	0.184	*0.5331*
tdT^+^ cells	CD45^+^ tdT^+^	0.5133	0.174	0.0024	0.001	***<0.0001***	0.0042	0.007	***<0.0001***
Neutrophils	CD45^+^ TCRβ^−^ CD19^−^ Gr1^hi^ CD11b^hi^	19.03	9.178	14.16	5.145	*0.3562*	14.8	9.194	*0.4710*
Eosinophils	CD45^+^ TCRβ^−^ CD19^−^ Neutro^−^ Macro^−^ CD11b^+^ SSC^hi^	1.465	0.765	1.585	0.655	*0.9235*	1.219	0.754	*0.7306*
**Peritoneal cavity**									
Mast cells	CD45^+^ F4/80^−^ FcϵRIα^+^ CD117^+^	1.746	0.724	1.895	0.677	*0.8788*	2.236	0.803	*0.2502*

Proportions of immune cell populations in the indicated compartments were determined by flow cytometry. Cells were defined following the indicated gating strategy; 15- to 25-week-old mice were analyzed. Statistical analysis was done by one-way analysis of variance (ANOVA) followed by Tukey’s multiple comparison test. p-values indicated are from comparison to the Mcpt8^CT/+^ group. SD, standard deviation.

Bold/Italic values in Table 1 and 2 are the statistically significant values (p<0.05).

### Confocal Microscopy

Mouse splenocytes were harvested as described above and resuspended in PBS containing 2% FCS and 2 mM of EDTA. Splenocytes were then depleted from B, NK, and T cells by magnetic selection through the use of biotinylated anti-CD19 (6D5), anti-NK1.1 (PK136), anti-CD8α (53-6.7), anti-CD4 (RM4-5) (BioLegend) and MagniSort™ streptavidin-negative selection beads, following manufacturer’s instructions (ThermoFisher Scientific, Waltham, MA, USA). This basophil-enriched cell suspension was then stained with a FITC-conjugated anti-IgE antibody (23G3, Southern Biotech, Birmingham, AL, USA) in FACS buffer, washed, incubated on poly-l-lysine-coated coverslips for 20 min at 37°C, washed in PBS, fixed with a fixation buffer (BioLegend), and permeabilized with PZB. The nuclei were stained with 360 nM of 4′,6-diamidino-2-phenylindole (DAPI) in PZB and washed in PBS. Coverslips were then mounted in Shandon Immu-Mount (ThermoFisher Scientific, Waltham, MA, USA) on superfrost slides (VWR, Radnor, PA, USA) and incubated overnight at 4°C. Image acquisition was realized with a Zeiss, Oberkochen, Germany LSM 780 confocal microscope, and image analysis was done with the Zen 2012 (Blue edition) service pack 2 software.

### *Ex Vivo* Primary Cell Stimulation

Mouse splenocytes were harvested through mechanical disruption over a 40-μm of cell strainer, and red blood cells were lysed as described above. Single-cell suspensions at 2 million cells/ml were cultured in a culture medium (RPMI 1640) with Glutamax and 20 mM of HEPES, 1 mM of Na-pyruvate, and 1× nonessential amino acids (all from Life Technologies); 100 μg/ml of streptomycin and 100 U/ml of penicillin (GE Healthcare, Chicago, IL, USA); and 37.5 μM of β-mercaptoethanol (Sigma-Aldrich, Burlington, MA, USA) supplemented with 20% heat-inactivated fetal calf serum (FCS) (Life Technologies) at 37°C and 5% CO_2_. For phorbol-myristate-acetate (PMA) and ionomycin-stimulation experiments, whole splenocytes were stimulated or not with 40 nM of PMA and 800 nM of ionomycin for 4 h in the presence of 2 µg/ml of brefeldin A (all from Sigma-Aldrich, Merck). For other stimulations, splenocytes were enriched in basophils by depleting T, B, and NK cells as described in the confocal microscopy section. CD200R1 upregulation was measured after 2 h of stimulation with 500 ng/ml of anti-IgE antibody (clone RME-1, BioLegend). Anti-IgE-induced IL-4 and IL-6 productions by basophils were quantified by flow cytometry after 4 h of stimulation with 500 ng/ml of anti-IgE antibody in the presence of brefeldin A. At the end of these stimulations, cells were harvested and treated as described in the *Flow Cytometry* section. For IL-3-induced basophil stimulation, cells were stimulated over 24 h with 1 ng/ml of recombinant mouse IL-3 (PeproTech, Rocky Hill, NJ, USA) and IL-4 and IL-6 productions were quantified by ELISA (R&D systems, Minneapolis, MN, USA), normalized to the number of basophils present in each well and expressed in picograms per 1,000 basophils.

### Mcpt8 mRNA qPCR Analysis

Single-cell suspensions of bone marrow (BM) cells (in PBS containing 2% FCS and 2 mM of EDTA) were stained with biotin-conjugated anti-Ly6G (1A8) and anti-CD19 (6D5) antibodies (BioLegend, San Diego, CA, USA) before removing positive cells with MagniSort™ streptavidin-negative selection beads following the manufacturer’s instructions (ThermoFisher Scientific, Waltham, MA, USA). After this basophil enrichment, primary BM basophils were selected for CD200R3 expression and sorted using a BD FACSMelody™ cell sorter (BD Biosciences, Franklin Lakes, NJ, USA). RNA extraction was performed by using Trizol reagent as described in the manufacturer’s protocol (Invitrogen). cDNA was synthesized with SuperScript™ III First-Strand Synthesis System (Invitrogen). Quantitative PCR was performed with SsoAdvanced SYBR green reaction mix (Bio-Rad, Hercules, CA, USA) using the M_B2m_1 KiCqStart™ primer pair for mouse β2-microglobulin as housekeeping gene (Sigma-Aldrich, Merck) and the following primers for Mcpt8 quantification: Forward primer: 5′-GTGGGAAATCCCAGTGAGAA-3′ and Reverse primer: 5′-TCCGAATCCAAGGCATAAAG-3′ (12). Quantitative PCR was performed on the CFX96 Touch Real-Time PCR Detection System (Bio-Rad, Hercules, CA, USA), and, following amplification, Ct values were obtained using the CFX Manager™ software 2.1 (Bio-Rad, Hercules, CA, USA).

### TH2 Polarization of Spleen CD4+ T Cells *Ex Vivo*

F(ab’)2 anti-CD3 (clone145-2C11 at 10 µg/ml) and anti-CD28 (clone PV-1 at 2 µg/ml) antibodies (BioXcell, Lebanon, NH, USA) were coated overnight at 4°C in 96-well plates (Costar) in sterile-filtered 20-mM carbonate buffer (pH 9.6) containing 2 mM of MgCl_2_ and 0.01% NaN_3_. Wells were washed in PBS before plating the cells. Total spleen CD4^+^ T cells were sorted by magnetic negative selection following the manufacturer’s instructions (STEMCELL Technologies, Vancouver, Canada). CD4^+^ T cells were then resuspended in a culture medium (same as above) at 0.25 million cells/ml. Two hundred microliters of cell suspension were then added to wells and supplemented with 10 ng/ml of IL-2 and 50 ng/mL of IL-4 (both from BioLegend, San Diego, CA, USA). After 2 days in the culture at 37°C and 5% CO_2_, IL-2 and IL-4 were added again at the same concentrations for 3 more days. TH2-polarized CD4^+^ T cells were then stimulated for 4 h with 40 nM of PMA and 800 nM of ionomycin in the presence of 2 µg/ml of brefeldin A (Sigma-Aldrich, Merck). Flow cytometry analysis was then performed as described above.

### Detection of Anti-RNP Autoantibodies

Maxisorp plates (Thermo Scientific) were coated overnight at 4°C with 10 µg/ml of purified RNP complexes (Immunovision, Springdale, AR, USA) diluted in carbonate buffer (100 mM of NaHCO_3_ and 30 mM of Na_2_CO_3_, pH 9.6). Plates were washed 3 times in 300 µl of PBS containing 0.05% Tween-20 (PBS-T, Bio-Rad Laboratories) and then saturated for 1 h at room temperature with 200 µl of PBS containing 5% FCS (Gibco, Waltham, MA, USA). Plates were then washed 3 times in 300 µl of PBS-T. All plasma samples were diluted 1:100 in PBS-T containing 5% FCS and 100 µl added to the wells and incubated for 2 h at room temperature. After 5 washes in PBS-T, a secondary antibody goat anti-mouse IgG conjugated to horseradish peroxidase (HRP) (Invitrogen) was diluted in PBS-T at 5% FCS at a final concentration of 500 ng/ml, and 100 µl was added to the wells for 1 h at room temperature. The plates were finally washed 5 times in 300 µl of PBS-T. A total of 100 µl of tetramethylbenzidine (TMB) substrate (ThermoFisher Scientific, Waltham, MA, USA) was added to the wells, incubated at room temperature for at least 20 min, and the reaction was stopped by adding 100 µl of 0.2 N of sulfuric acid solution. Optical density at 450 nm was measured by spectrophotometry (Infinite 200 Pro plate reader, Tecan, Männedorf, Germany). On each plate, similar negative and positive controls were run. Optical density (OD) values were first normalized to the negative controls, and then, the presented results were normalized to the mean of the PBS-injected control mice values and expressed in arbitrary units.

### Kidney Immunofluorescence Assays

Acetone-fixed 4 µm cryosections of OCT-embedded kidneys were thawed and blocked in 10% fetal calf serum (FCS) (Gibco, Waltham, MA, USA). Slices were stained with FITC-conjugated anti-mouse C3 (Cedarlane, Burlington, ON, Canada) or Alexa Fluor^®^ 488-conjugated anti-mouse IgG F(ab)’2 (Jackson Immunoresearch, West Grove, PA, USA), or their respective isotype controls, before being mounted in Immunomount (ThermoFisher Scientific, Waltham, MA, USA) and analyzed by fluorescent microscopy (Leica DMR, Leica Microsystems, Wetzlar, Germany). The ratio of specific glomerular fluorescence over tubulointerstitial background was then measured using ImageJ software (NIH), averaging 30 glomeruli per mouse for each sample.

### Statistics

Student’s unpaired *t*-tests were used to compare the differences of one variable between two groups when distributions were Gaussian and Mann–Whitney *U* tests for nonparametric distributions. When more than two groups were compared, one-way ANOVA coupled with Tukey’s multiple comparisons test was used. Statistics were done using Prism v9 software (Graphpad).

## Results

### CT-M8: A New Basophil-Specific Mouse Model

We aimed at developing a basophil-specific mouse model in which we could both follow basophil localization by immunofluorescence and flow cytometry and express the CRE recombinase selectively in the basophil compartment. To achieve that goal, we took advantage of the gene encoding for the basophil-specific protease Mcpt8 (8). The strategy described in the *Methods* section and **Figure 1A** led to the generation of *Mcpt8^tm1Ics^* mice named here “CT-M8” (*Mcpt8^CT/CT^*), standing for CRE recombinase and tdTomato expressions (CT) under the control of *Mcpt8* gene transcription (M8). CT-M8 mice had normal fertility and did not show any dysregulation of their immune cell distribution in lymphoid organs or blood (**Table 1**). As expected, the construct allowed straightforward basophil detection by flow cytometry (**Figure 1B**). Direct tdTomato^+^ basophil gating on living singlet CD45^+^ cells was as efficient to detect basophils as a conventional gating strategy for basophils (CD45^lo^ CD49b^+^ FcϵRIα^+^ CD123^+^ CD200R3^+^) (**Figure 1B**; **Table 1**). By confocal microscopy, basophil detection was evident in CT-M8 mice, and tdTomato expression in the spleen was restricted to IgE-bearing cells (**Figure 1C**; **Supplementary Figure S1**). Of note, tdTomato expression in *Mcpt8^CT/+^* basophils was half that in *Mcpt8^CT/CT^* basophils (**Figures 1B, D**). Although inserted after the stop codon, the construct inserted into the *Mcpt8* locus led to a drastic reduction of *Mcpt8* mRNA expression as measured by qPCR in bone marrow (BM) basophils isolated from *Mcpt8^CT/CT^* mice. However, *Mcpt8* mRNA expression was still significantly retained in *Mcpt8^CT/+^* basophils (**Figure 1E**).

**Figure 2:**
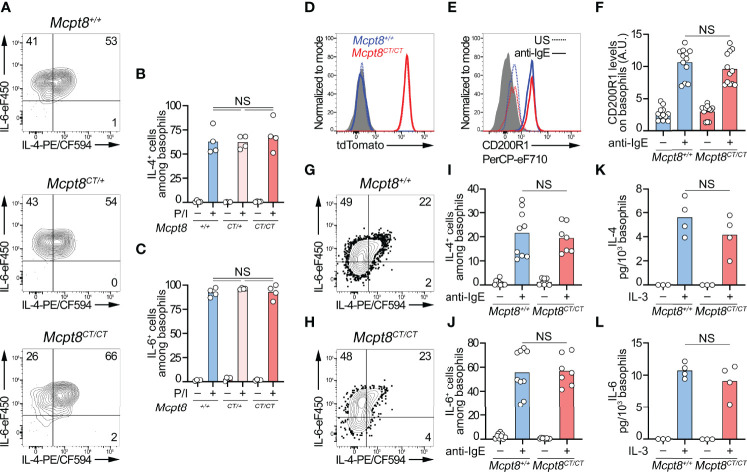
CT-M8 mice have fully functional basophils. **(A–C)** Splenocytes from *Mcpt8^+/+^*, *Mcpt8^CT/+^*, and *Mcpt8^CT/CT^* mice were stimulated over 4 h with PMA and ionomycin (P/I) in the presence of brefeldin A. Proportions of IL-4^+^ and IL-6^+^ basophils were assessed by intracellular flow cytometry staining as depicted in **(A)** and quantified in **(B, C)** (see *Methods*). (**D**–**F**) Splenocytes (depleted of T, B, and NK cells) from *Mcpt8^+/+^* (blue) and *Mcpt8^CT/CT^* (red) mice were left unstimulated (US, dotted line) or were stimulated over 2 h with 0.5 µg/ml of anti-IgE antibody (solid line). tdTomato **(D)** and activation marker CD200R1 expression levels **(E)** were assessed in basophils by flow cytometry. Quantification of CD200R1 expression levels on basophils is shown in **(F)**. **(G–J)** T-, B-, and NK-cell-depleted splenocytes from *Mcpt8^+/+^* (blue) and *Mcpt8^CT/CT^* (red) mice were stimulated over 4 h with anti-IgE antibody (anti-IgE) in the presence of brefeldin A (see *Methods*). As depicted in **(G, H)**, proportions of IL-4^+^ **(I)** and IL-6^+^ **(J)** basophils were assessed by flow cytometry. **(K, L)**. The same cells as in **(G–J)** were stimulated (+) or not (−) over 24 h with 1 ng/ml of mouse IL-3 without brefeldin A (see *Methods*). IL-4 and IL-6 concentrations in the culture supernatant were measured by ELISA and normalized to the number of basophils in each condition. Statistical analyses were done by one-way ANOVA followed by Tukey’s multiple comparison tests between the indicated groups. NS, not significant (*p* > 0.05). **(A–J)** Results from at least 3 different mice per group from 3 independent experiments are shown. **(K**, **L)** Results from 3 different mice are shown.

tdTomato expression was strictly restricted to the basophil population in all tested organs (blood, peritoneal lavage, lymph nodes, bone marrow, and spleen) (**Figure 1D**; **Table 1**). Spleen basophils from *Mcpt8^CT/CT^* and *Mcpt8^CT/+^* mice produced IL-4 and IL-6 to the same extent as WT (*Mcpt8^+/+^*) basophils after phorbol myristate acetate (PMA) and ionomycin stimulation, demonstrating that construct expression did not alter the maximal abilities of the basophils from CT-M8 mice to produce these cytokines (**Figures 2A–C**). CD200R1 is a recognized mouse basophil activation marker (13, 14). Anti-IgE-mediated basophil activation resulted in similar CD200R1 upregulation on spleen basophils from *Mcpt8^+/+^* and *Mcpt8^CT/CT^* mice and to similar IL-4 and IL-6 productions (**Figures 2D–J**). Furthermore, IL-3-mediated activation was as efficient in *Mcpt8^+/+^* as in *Mcpt8^CT/CT^* basophils to induce IL-4 and IL-6 productions (**Figures 2K, L**).

Overall, these data demonstrated that the CT-M8 mice (i) allowed convenient and selective basophil detection by microscopy and flow cytometry, (ii) did not show any abnormalities in their immune cell populations, and (iii) had a tdTomato expression restricted to basophils in all the compartments analyzed, and that basophils from these mice, despite an alteration of Mcpt8 expression, exhibited normal distribution and normal function as assessed after IgE-, IL-3-, and PMA-ionomycin-mediated stimulations.

### CRE Expression in CT-M8 Mice Is Basophil-Specific and Functional

**Figure 3:**
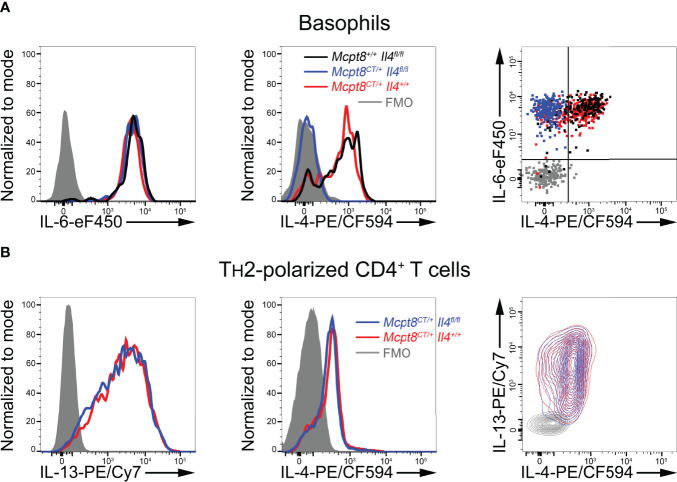
Functional and basophil-specific CRE recombinase expression in CT-M8 mice. **(A)** Splenocytes from *Mcpt8^+/+^ Il4^fl/fl^* (black lines/dots), *Mcpt8^CT/+^ Il4^+/+^* (red lines/dots), and *Mcpt8^CT/+^ Il4^fl/fl^* (blue lines/dots) were stimulated for 4 h with PMA and ionomycin in the presence of brefeldin A (see *Methods*). IL-6 (left) and IL-4 (middle) productions were assessed in basophils by intracellular flow cytometry staining. Productions are also shown together on the right panel as a dot plot. Gray-filled histograms and gray dots represent signals from basophils intracellularly incubated with fluorophore-conjugated isotype controls (fluorescence minus one (FMO)). **(B)** TH2-polarized CD4^+^ T cells from mice with the indicated genotypes (see *Methods*) were stimulated with PMA and ionomycin for 4 h in the presence of brefeldin **(A)** Productions were assessed by intracellular staining of IL-13 (left) and IL-4 (middle). Productions are also shown together on the right panel as a contour plot. **(A, B)** Representative data from one mouse per genotype are shown. A minimum of 3 mice per genotype were analyzed, showing similar results.

We next sought to verify that functional CRE recombinase expression occurred only in basophils and allowed induction of basophil-specific floxed gene recombination. To that end, *Mcpt8^CT/+^* mice were bred with *Il4^fl/fl^* mice (11). Unlike IL-6 production, which was not altered, PMA-ionomycin-induced IL-4 production by basophils from *Mcpt8^CT/+^ Il4^fl/fl^* mice was completely abrogated as compared to *Mcpt8^CT/+^ Il4^+/+^* and *Mcpt8^+/+^ Il4^fl/fl^* mice (**Figure 3A**). Thus, CRE recombinase was functional, and its expression was restricted to the basophil compartment since IL-4 production in *in vitro* polarized T helper type 2 (TH2) CD4^+^ T cells was not impaired in *Mcpt8^CT/+^ Il4^fl/fl^* mice as compared to *Mcpt8^CT/+^ Il4^+/+^* mice (**Figure 3B**).

**Figure 4:**
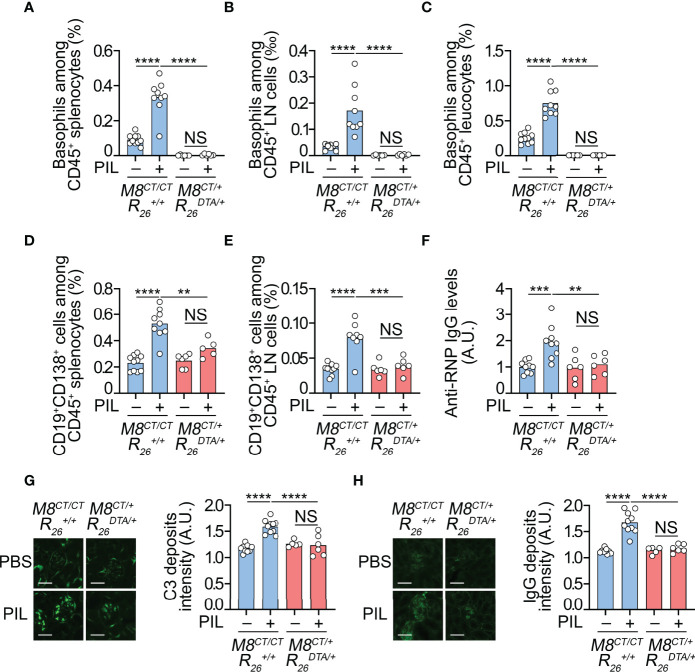
Basophils have a nonredundant role in PIL development. **(A**–**H)** Eight-week-old *Mcpt8^CT/CT^ Rosa26^+/+^* (basophil sufficient, *M8^CT/CT^* $R_{26}^{+/+}$ ) and *Mcpt8^CT/+^ Rosa26^DTA/+^* (basophil deficient, *M8^CT/+^* $R_{26}^{DTA/+}$ ) mice were injected ip with PBS or pristane (PIL). Eight weeks later, the mice were euthanized and analyzed. **(A–C)** Basophil accumulation in the spleen **(A)** and peripheral lymph nodes (LNs) (cervical, axillary, and inguinal) **(B)** and blood basophils **(C)** were quantified by flow cytometry, as shown in **Figure 1** and described in the *Methods* section. Basophils were defined as CD45^lo^ CD3^−^ CD19^−^ CD117^−^ CD49b^+^ FcϵRIα^+^ tdTomato^+^ cells among CD45^+^ viable singlets. **(D, E)** CD19^+^ CD138^+^ plasma cells were quantified in the spleen **(D)** and peripheral lymph nodes **(E)** of the mice described above. **(F)** Anti-RNP IgG autoantibody titers were quantified by ELISA from plasma samples of indicated mice, as described in the *Methods* section. The O.D. values at 450 nm were determined, and data were normalized to the mean of PBS-injected *Mcpt8^CT/CT^ Rosa26^+/+^* values. A.U., arbitrary units. **(G, H)** Four-micrometer cryosections of kidneys were analyzed by immunofluorescence for C3 **(G)** and IgG **(H)** staining as shown on images (scale bar, 60 µm) and quantified in adjacent bar graphs as A.U., corresponding to the ratio between fluorescence intensity measured in glomeruli to that in interstitial background. **(A**–**H)** Results are from at least three independent experiments and are presented as individual values in bars representing the mean values. Statistical analyses were done by one-way ANOVA followed by Tukey’s multiple comparison tests between the indicated groups. NS, not significant, *p* > 0.05; ^**^ *p* < 0.01; ^***^ *p* < 0.001; ^****^ *p* < 0.0001.

Next, we bred the CT-M8 mice with the ROSA-DTA mice (9). *Mcpt8^CT/+^ Rosa26^DTA/+^* mice were constitutively basophil-deficient and did not show any other immune cell population defects in homeostatic conditions as compared to *Mcpt8^CT/+^ Rosa26^+/+^* and *Mcpt8^+/+^ Rosa26^DTA/DTA^* mice in all analyzed compartments (**Table 2**; **Figures 4A–C**).

Overall, these results confirmed the basophil-restricted expression of a functional CRE recombinase in CT-M8 mice that allowed generating basophil-specific ablation of selected genes and a constitutive basophil-deficient mouse model.

### Basophils Have a Nonredundant Role in Lupus-Like Nephritis Amplification

By accumulating in SLOs during lupus pathogenesis, we previously showed that basophils support the survival of autoreactive antibody-secreting cells, leading to the amplification of autoantibody titers both in lupus-like mouse models and in SLE patient cohorts (4, 6, 7). Pristane (TMPD; 2, 6, 10, 14- tetramethylpentadecane) is a mineral oil able to induce the development of a lupus-like phenotype when injected intraperitoneally (ip) into C57BL/6 mice (15). The resulting PIL was associated with peripheral basophilia and an accumulation of basophils in SLOs. When depleting basophils using different approaches over 14 days in C57BL/6 female mice in established PIL (24 weeks after pristane injection), the accumulation of autoantibody-secreting plasma cells and amplification of autoantibody production were dampened (7). However, these approaches did not allow for evaluating the contribution of basophils to the onset of the disease.

We, therefore, took advantage of our new model of constitutive basophil-deficient mice described above to address this question. Basophil-sufficient (*Mcpt8^CT/CT^ Rosa26^+/+^*) and basophil-deficient (*Mcpt8^CT/+^ Rosa26^DTA/+^*) female mice were injected ip with pristane at around 8 weeks of age and euthanized for analysis 8 weeks after injection. This time point corresponded to an early stage of PIL since neither proteinuria nor other biological parameters evidencing kidney dysfunction (blood urea nitrogen and creatininemia) were detectable (*data not shown*). However, 8 weeks after pristane injection in basophil-sufficient animals, basophil and short-lived plasma cell (CD19^+^CD138^+^ B cells) accumulation in SLOs (spleen and lymph nodes), peripheral basophilia, and autoantibodies in plasma were detected (**Figures 4A–F**). This was associated with the detection of complement component C3 and IgG deposits in the kidney glomeruli of pristane-injected mice, unlike the glomeruli of PBS-injected control mice, confirming the early development stages of the pristane-induced lupus-like nephritis (**Figures 4G, H**).

As expected, no basophil was detected in basophil-deficient mice (*Mcpt8^CT/+^ Rosa26^DTA/+^*) 8 weeks after pristane injection in any of the compartments analyzed (**Figures 4A–C**). No accumulation of CD19^+^CD138^+^ short-lived plasma cells was detected in SLOs of pristane-injected basophil-deficient mice, suggesting that basophil deficiency prevented PIL development (**Figures 4D, E**). Indeed, no significant increase in antiribonucleoprotein (anti-RNP) antibodies, autoantibodies present in PIL in C57BL/6 mice (6, 7), was measurable in the blood of basophil-deficient mice (**Figure 4F**). Moreover, no significant C3 and IgG deposits in the glomeruli of basophil-deficient mice were detected 8 weeks after pristane injection (**Figures 4G, H**).

Overall, these results demonstrate that basophils have a nonredundant role in PIL development as basophil deficiency sufficed to prevent the onset of autoantibody production and lupus-like nephritis induced by pristane injection.

## Discussion

Beyond its role in allergic and parasitic diseases, type 2 immunity is now identified as a key player in the pathophysiology of various autoimmune diseases (16, 17). Several studies have demonstrated its relevance to SLE pathogenesis and identified IgE and basophils as promising therapeutic targets due to their role in amplifying both autoantibody production and tissue damage. Most of these studies are based on a translational approach that allowed us to study and decipher in lupus-like mouse models what was observed in human patients (4, 6, 18–20). The use of a novel mouse model allowing basophil-specific short-term depletion (*Mcpt8^DTR^* mice (21)) demonstrated that basophil targeting efficiently dampened lupus-like activity in mice with established disease (6). Here, using a new basophil-deficient mouse model, we show that basophils were required to reach a pathogenic threshold in PIL, supporting their nonredundant role in early lupus-like disease development.

Besides specific depletion of basophils, the new CT-M8 mouse model also enables convenient detection of basophils by flow cytometry and confocal microscopy as well as the selective depletion of a floxed gene in the basophil compartment. In particular, our analysis showed that constitutive basophil deficiency in this model did not lead to any identified immunological bias. Likewise, mice rendered IL-4 deficient in the basophil compartment did not impact the ability of TH2-polarized CD4^+^ T cells from the same mice to produce IL-4. Unexpectedly, *Mcpt8* mRNA expression was dramatically dampened by the insertion of the IRES-based construct after the stop codon site of the Mcpt8 gene. If a knock-in approach would be expected to induce Mcpt8 deficiency (22), the reason explaining this alteration of Mcpt8 expression in our model will need further investigation. Fortunately, Mcpt8 expression was still detected in *Mcpt8^CT/+^* heterozygous mice, allowing for the generation of Mcpt8-sufficient mice expressing both tdTomato and CRE recombinase only in the basophil compartment. Of note, Mcpt8-reduced expression is known not to induce any significant alteration in basophil function in mouse models of parasite infection (22) or sepsis (12).

To our knowledge, three other mouse models expressing the CRE recombinase selectively in basophils are currently available. Generated in 2010 by Voehringer and colleagues, the “Mcpt8CRE” mouse is a BAC transgenic mouse strain expressing the Cre recombinase inserted behind the start codon of the Mcpt8 gene. Unfortunately, these mice showed a constitutive 90% basophil deficiency, probably due to CRE overexpression preventing its use for floxed gene deletion selectively in basophils (23). The “Basoph8” mice were developed by Locksley and colleagues, who replaced the Mcpt8 gene at the start site by inserting a reporter cassette containing a sequence encoding the yellow fluorescent protein (YFP), followed by an IRES and a sequence encoding humanized Cre recombinase. This mouse strain is effective at basophil-specific Cre-mediated gene deletion and expresses constitutively the YFP in basophils (22). The third one was recently developed by Karasuyama and colleagues and called *Mcpt8^iCre/+^*. This mouse strain expresses the improved Cre (iCre) recombinase inserted to replace the first exon of the Mcpt8 gene and is efficient in basophil-specific Cre-mediated gene deletion but does not express constitutively any fluorescent reporter gene (11).

The advantages of the CT-M8 mice are then similar to the ones of the Basoph8 mouse model (22), but they express another fluorescent protein (tdTomato), allowing distinct breeding strategies for the generation of multiple gene/cell-specific reporter mouse strains. In addition, the CT-M8 mice (in their heterozygous *Mcpt8^CT/+^* strain) retain significant expression of the *Mcpt8* gene while allowing fully sufficient functional expression of the CRE recombinase.

Mast cells and basophils share immediate common progenitors, and Mcpt8 gene-controlled CRE or DTR expression may be detected at low levels in other cell types including mast cells, granulocyte-macrophage progenitors, T cells, B cells, NK cells, eosinophils, and dendritic cells (23–25). Therefore, both the construct used to generate CT-M8 mice and DTα expression in Mcpt8-expressing cells could have affected immune cell populations other than basophils. However, analysis of CT-M8 mice showed that tdTomato expression was fully restricted to the basophil compartment (**Table 1**). As well, Mcpt8-dependent DTα expression in *Mcpt8^CT/+^ R_26_-Stop^fl/WT^-DTA* mice did not impact any immune cell populations other than basophils (**Table 2**). The very low Mcpt8 mRNA expression levels in the former cell populations, or their progenitors, as compared to mature basophils, may explain the basophil-selectivity observed in the various Mcpt8 gene-based mouse models.

In the present study, basophil deficiency prevented the development of the pristane-induced lupus-like disease. Indeed, our data suggest that basophils are necessary to reach a pathogenic threshold in early PIL. This is evidenced by the ablation of plasmablast accumulation in SLOs, autoantibody production, and glomerular deposition of circulating immune complexes in basophil-deficient mice.

Further investigations will be required to determine the mechanisms by which basophils contribute to autoimmune evolution in the PIL. In this context, our new basophil-specific mouse model will allow selective depletion in the basophil compartment of candidate genes. For instance, it will allow deciphering of the axis of autoreactive IgE and FcϵRI-mediated basophil activation in the nonredundant role of basophils in lupus-like pathogenesis. Indeed, autoreactive IgE is highly prevalent in SLE patients with active disease (26), and the basophil activation status correlates strongly with disease activity (4, 6). Furthermore, IgE itself is known to exert an immunoregulatory role in various lupus-like mouse models (4, 18). We will now be able to deplete specifically FcϵRI in basophils by breeding CT-M8 mice with *FcϵRIγ^fl/fl^* mice (C57BL/6J-Fcer1g^em1Gfng/J^, *Jax* mice #036592) and demonstrate whether basophil involvement in lupus-like disease amplification depends on FcϵRI-mediated signals. The role of factors produced by basophils during the course of the disease, such as IL-4 or IL-6, could be assessed as well. Although technically challenging, basophil reconstitution of basophil-deficient animals through frequent adoptive transfer of purified basophils during the whole procedure may further help to decipher the non-redundant role of basophils in PIL onset.

The nonredundant role of basophils in lupus-like pathogenesis demonstrated in PIL will need to be validated in other lupus-like mouse models, including spontaneous models. This preclinical development is necessary to determine whether targeting basophils or basophil-activating factors in recently diagnosed SLE patients may be a promising therapeutic strategy to prevent the development of lesions in targeted organs. Several approaches may be envisaged to alter the basophil contribution to lupus nephritis development, including targeting IgE, PTGDRs, or directly basophils through a basophil-specific surface marker that still needs to be identified. Basophils have a deleterious role in various allergic, inflammatory, and autoimmune diseases (16, 27). Identifying therapeutic strategies targeting these cells, their mediators, or the factors involved in their contribution to these diseases may represent a promising area of clinical development.

## Data Availability Statement

The raw data supporting the conclusions of this article will be made available by the authors, without undue reservation.

## Ethics Statement

The animal study was reviewed and approved by the comité d’éthique Paris Nord N°121 and the Ministère de l’enseignement supérieur, de la recherche et de l’innovation under the authorization number APAFIS#14115.

## Author Contributions

JT designed the experiments, conducted the experiments, and wrote the manuscript. NC conceived the project, designed the experiments, conducted the experiments, wrote the manuscript, and directed the project. QS, LC, CP, UB, MB, and ED conducted the experiments, analyzed the data, and/or edited the manuscript. HK and KM provided the *Il4^fl/fl^* mice and edited the manuscript. NC had full access to all of the data in the study and takes responsibility for the integrity of the data and the accuracy of the data analysis. All authors listed have made a substantial, direct, and intellectual contribution to the work and approved it for publication.

## Funding

This work was supported by the Fondation pour la Recherche Médicale (FRM) (grant # EQU201903007794) to NC, the French Agence Nationale de la Recherche (ANR) (grants # ANR-19-CE17-0029 BALUMET to NC and ANRPIA-10-LABX-0017 INFLAMEX), by the Centre National de la Recherche Scientifique (CNRS), by Université de Paris, and by the Institut National de la Santé et de la Recherche Médicale (INSERM).

## Conflict of Interest

CP and NC are coinventors of the patent WO2016128565A1 related to the use of PTGDR-1 and PTGDR-2 antagonists for the prevention or treatment of systemic lupus erythematosus. NC holds a patent related to compositions and methods for treating or preventing lupus (W020120710042).

The remaining authors declare that the research was conducted in the absence of any commercial or financial relationships that could be construed as a potential conflict of interest.

## Publisher’s Note

All claims expressed in this article are solely those of the authors and do not necessarily represent those of their affiliated organizations, or those of the publisher, the editors and the reviewers. Any product that may be evaluated in this article, or claim that may be made by its manufacturer, is not guaranteed or endorsed by the publisher.

## References

[1] Anders HJ Saxena R Zhao MH Parodis I Salmon JE Mohan C. Lupus Nephritis. Nat Rev Dis Primers (2020) 6(1):7. doi: 10.1038/s41572-019-0141-9 31974366

[2] Dema B Charles N. Autoantibodies in SLE: Specificities, Isotypes and Receptors. Antibod (Basel) (2016) 5(1):2. doi: 10.3390/antib5010002 PMC6698872 31557984

[3] Dema B Charles N. Advances in Mechanisms of Systemic Lupus Erythematosus. Discovery Med (2014) 17(95):247–55. 24882716

[4] Charles N Hardwick D Daugas E Illei GG Rivera J. Basophils and the T Helper 2 Environment can Promote the Development of Lupus Nephritis. Nat Med (2010) 16(6):701–7. doi: 10.1038/nm.2159 PMC2909583 20512127

[5] Charles N Watford WT Ramos HL Hellman L Oettgen HC Gomez G. Lyn Kinase Controls Basophil GATA-3 Transcription Factor Expression and Induction of Th2 Cell Differentiation. Immunity (2009) 30(4):533–43. doi: 10.1016/j.immuni.2009.02.008 PMC2772996 19362019

[6] Pellefigues C Dema B Lamri Y Saidoune F Chavarot N Loheac C. Prostaglandin D2 Amplifies Lupus Disease Through Basophil Accumulation in Lymphoid Organs. Nat Commun (2018) 9(1):725. doi: 10.1038/s41467-018-03129-8 29463843 PMC5820278

[7] Dema B Lamri Y Pellefigues C Pacreau E Saidoune F Bidault C. Basophils Contribute to Pristane-Induced Lupus-Like Nephritis Model. Sci Rep (2017) 7(1):7969. doi: 10.1038/s41598-017-08516-7 28801578 PMC5554199

[8] Poorafshar M Helmby H Troye-Blomberg M Hellman L. MMCP-8, the First Lineage-Specific Differentiation Marker for Mouse Basophils. Elevated Numbers of Potent IL-4-Producing and MMCP-8-Positive Cells in Spleens of Malaria-Infected Mice. Eur J Immunol (2000) 30(9):2660–8. doi: 10.1002/1521-4141(200009)30:9<2660::AID-IMMU2660>3.0.CO;2-I 11009100

[9] Voehringer D Liang HE Locksley RM. Homeostasis and Effector Function of Lymphopenia-Induced "Memory-Like" T Cells in Constitutively T Cell-Depleted Mice. J Immunol (2008) 180(7):4742–53. doi: 10.4049/jimmunol.180.7.4742 PMC2670614 18354198

[10] Birling MC Dierich A Jacquot S Herault Y Pavlovic G. Highly-Efficient, Fluorescent, Locus Directed Cre and FlpO Deleter Mice on a Pure C57BL/6N Genetic Background. Genesis (2012) 50(6):482–9. doi: 10.1002/dvg.20826 22121025

[11] Shibata S Miyake K Tateishi T Yoshikawa S Yamanishi Y Miyazaki Y. Basophils Trigger Emphysema Development in a Murine Model of COPD Through IL-4-Mediated Generation of MMP-12-Producing Macrophages. Proc Natl Acad Sci U.S.A. (2018) 115(51):13057–62. doi: 10.1073/pnas.1813927115 PMC6305004 30510003

[12] Piliponsky AM Shubin NJ Lahiri AK Truong P Clauson M Niino K. Basophil-Derived Tumor Necrosis Factor can Enhance Survival in a Sepsis Model in Mice. Nat Immunol (2019) 20(2):129–40. doi: 10.1038/s41590-018-0288-7 PMC6352314 30664762

[13] Bakocevic N Claser C Yoshikawa S Jones LA Chew S Goh CC. CD41 is a Reliable Identification and Activation Marker for Murine Basophils in the Steady State and During Helminth and Malarial Infections. Eur J Immunol (2014) 44(6):1823–34. doi: 10.1002/eji.201344254 24610714

[14] Torrero MN Larson D Hubner MP Mitre E. CD200R Surface Expression as a Marker of Murine Basophil Activation. Clin Exp Allergy (2009) 39(3):361–9. doi: 10.1111/j.1365-2222.2008.03154.x PMC2743132 19134017

[15] Reeves WH Lee PY Weinstein JS Satoh M Lu L. Induction of Autoimmunity by Pristane and Other Naturally Occurring Hydrocarbons. Trends Immunol (2009) 30(9):455–64. doi: 10.1016/j.it.2009.06.003 PMC2746238 19699150

[16] Charles N. Autoimmunity, IgE and FcepsilonRI-Bearing Cells. Curr Opin Immunol (2021) 72:43–50. doi: 10.1016/j.coi.2021.03.003 33819742

[17] Maurer M Altrichter S Schmetzer O Scheffel J Church MK Metz M. Immunoglobulin E-Mediated Autoimmunity. Front Immunol (2018) 9:689. doi: 10.3389/fimmu.2018.00689 29686678 PMC5900004

[18] Dema B Charles N Pellefigues C Ricks TK Suzuki R Jiang C. Immunoglobulin E Plays an Immunoregulatory Role in Lupus. J Exp Med (2014) 211(11):2159–68. doi: 10.1084/jem.20140066 PMC4203948 25267791

[19] Henault J Riggs JM Karnell JL Liarski VM Li J Shirinian L. Self-Reactive IgE Exacerbates Interferon Responses Associated With Autoimmunity. Nat Immunol (2016) 17(2):196–203. doi: 10.1038/ni.3326 26692173 PMC4718782

[20] Pan Q Gong L Xiao H Feng Y Li L Deng Z. Basophil Activation-Dependent Autoantibody and Interleukin-17 Production Exacerbate Systemic Lupus Erythematosus. Front Immunol (2017) 8:348. doi: 10.3389/fimmu.2017.00348 28396669 PMC5366357

[21] Wada T Ishiwata K Koseki H Ishikura T Ugajin T Ohnuma N. Selective Ablation of Basophils in Mice Reveals Their Nonredundant Role in Acquired Immunity Against Ticks. J Clin Invest (2010) 120(8):2867–75. doi: 10.1172/JCI42680 PMC2912199 20664169

[22] Sullivan BM Liang HE Bando JK Wu D Cheng LE McKerrow JK. Genetic Analysis of Basophil Function *In Vivo* . Nat Immunol (2011) 12(6):527–35. doi: 10.1038/ni.2036 PMC3271435 21552267

[23] Ohnmacht C Schwartz C Panzer M Schiedewitz I Naumann R Voehringer D. Basophils Orchestrate Chronic Allergic Dermatitis and Protective Immunity Against Helminths. Immunity (2010) 33(3):364–74. doi: 10.1016/j.immuni.2010.08.011 20817571

[24] El Hachem C Hener P Kirstetter P Li J Chan S Li M. Treatment of MCPT8(DTR) Mice With High- or Low-Dose Diphtheria Toxin Leads to Differential Depletion of Basophils and Granulocyte-Macrophage Progenitors. Eur J Immunol (2018) 48(5):861–73. doi: 10.1002/eji.201747351 29315532

[25] Wanet A Bassal MA Patel SB Marchi F Mariani SA Ahmed N. E-Cadherin is Regulated by GATA-2 and Marks the Early Commitment of Mouse Hematopoietic Progenitors to the Basophil and Mast Cell Fates. Sci Immunol (2021) 6(56):eaba0178. doi: 10.1126/sciimmunol.aba0178 33547048 PMC8261706

[26] Dema B Pellefigues C Hasni S Gault N Jiang C Ricks TK. Autoreactive IgE is Prevalent in Systemic Lupus Erythematosus and is Associated With Increased Disease Activity and Nephritis. PloS One (2014) 9(2):e90424. doi: 10.1371/journal.pone.0090424 24587356 PMC3938730

[27] Karasuyama H Miyake K Yoshikawa S Yamanishi Y. Multifaceted Roles of Basophils in Health and Disease. J Allergy Clin Immunol (2018) 142(2):370–80. doi: 10.1016/j.jaci.2017.10.042 29247714

