# Supplementary material for: CT-M8 Mice: A New Mouse Model Demonstrates That Basophils Have a Nonredundant Role in Lupus-Like Disease Development
Source: Front Immunol. 2022 Jun 29;13:900532. doi: 10.3389/fimmu.2022.900532 (PMC9277511; doi:10.3389/fimmu.2022.900532)
Supplement: Supplementary file 1 [file DataSheet_1.pdf]

CT-M8 mice: a new mouse model demonstrates that basophils have a non-redundant role in lupus-like disease development

SUPPLEMENTARY MATERIAL

Authors: John TCHEN [1,2], Quentin SIMON [1,2], Léa CHAPART [1,2], Christophe PELLEFIGUES [1,2], Hajime KARASUYAMA [3], Kensuke MIYAKE [3], Ulrich BLANK [1,2], Marc BENHAMOU [1,2], Eric DAUGAS [1,2,4], Nicolas CHARLES [1,2]

Affiliations:

1- Université Paris Cité, Centre de Recherche sur l'Inflammation, INSERM UMR1149, CNRS EMR8252, Faculté de Médecine site Bichat, Paris, France

2- Université Paris Cité, Laboratoire d'Excellence INFLAMEX, Paris, France

3- Department of Immune Regulation, Graduate School of Medical and Dental Sciences, Tokyo Medical and Dental University (TMDU), Tokyo, Japan

4- Service de Néphrologie, Hôpital Bichat, Assistance Publique – Hôpitaux de Paris, Paris, France

*__Correspondence to__:
Nicolas Charles, PhD
Centre de Recherche sur l'Inflammation, INSERM UMR1149, CNRS ERL8252,
Université Paris Cité, Faculté de Médecine site Bichat,
16 rue Henri Huchard, 75018 Paris, France.
Phone: +33 157277306
E-mail: nicolas.charles@inserm.fr

Supplementary Figure S1 :

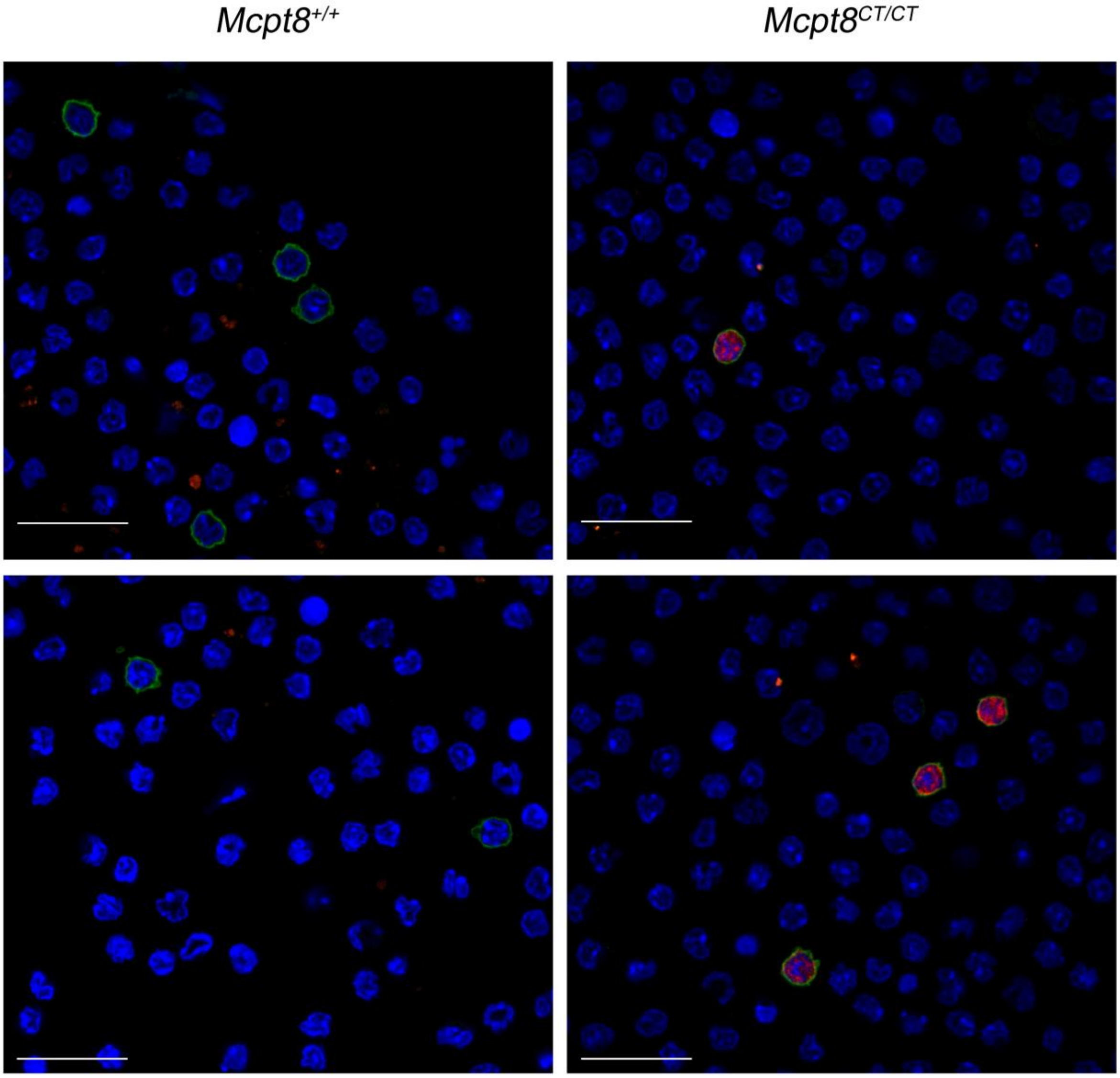

Supplementary Figure S1: tdTomato expression in basophils from CT-M8 mice

Basophil-specific tdTomato expression (red) imaged by confocal microscopy on basophil-enriched splenocytes from *Mcpt8*$^{+/+}$ (left) and *Mcpt8*$^{CT/CT}$ (right) mice stained with anti-IgE antibody (green) and DNA staining (DAPI, blue) (see **methods**). Scale bar = 20 µm. The upper panels correspond to the uncropped images from **Figure 1C**. The lower panels are from another independent experiment.
